# Pseudoxanthome élastique généralisé et évolutif chez une mélanoderme

**DOI:** 10.11604/pamj.2013.16.132.3316

**Published:** 2013-12-09

**Authors:** Jean Baptiste Andonaba, Fatou Barro-Traoré, Somé Korsaga, Boukary Diallo, Jean Wilfried Diallo, Adama Traoré

**Affiliations:** 1 Service de Dermatologie, Centre Hospitalier Universitaire Souro Sanou, Bobo-Dioulasso, Burkina Faso; 2 Institut Supérieur des Sciences de la Santé/Université Polytechnique de Bobo-Dioulasso, Burkina Faso; 3 Unite de Formation et de Recherche en Science De la Santé (UFR-SDS), Université de Ouagadougou, Burkina Faso; 4 Service de Dermatologie, Centre Hospitalier Universitaire Yalgado Ouédraogo, Ouagadougou, Burkina Faso; 5 Service d'Ophtalmologie, Centre Hospitalier Universitaire Souro Sanou, Bobo-Dioulasso, Burkina Faso

**Keywords:** Pseudoxanthome élastique, peau, rétine, Burkina Faso, elastic pseudoxanthoma, skin, retina, Burkina Faso

## Abstract

Le pseudoxanthome élastique (PXE) est une maladie héréditaire du tissu élastique affectant principalement la peau, les yeux et les artères. Nous rapportons une forme évolutive compliquée pour souligner la nécessité de la précocité du diagnostic et du suivi, de l'approche multidisciplinaire et la prise en charge des complications. Une femme de 51 ans, a consulté pour des nappes fripées de papules typiquement en « peau de chagrin » associées à une baisse de l'acuité visuelle, des stries angioïdes compliquées de néovaisseaux choroïdiens aux deux yeux. Les lésions cutanées étaient disséminées à tout le corps mais prédominaient aux plis et les régions adjacentes. Cette forme généralisée et compliquée serait due au retard au diagnostic et à la consultation, habituelle dans notre pays. Les atteintes extra cutanées font toute la gravité de cette maladie qui nécessite une surveillance à vie. Les diagnostics différentiels se font avec la peau citréine, la papulose cervicale fibro-élastique et les PXE exogènes. Aucun traitement spécifique n'est disponible à ce jour. Le PXE nécessite une approche multidisciplinaire pour un conseil génétique et pour sa prise en charge globale et précoce.

## Introduction

Le Pseudoxanthome Elastique (PXE) ou Elastorrhexie systématisée ou syndrome de Gronblad-Strandberg est une maladie héréditaire du tissu élastique affectant principalement la peau, les yeux, les artères et le coeur [1]. Il touche les deux sexes avec une prédominance féminine non expliquée. L'affection est caractérisée, sur le plan cutané, par l'apparition de plaques jaunâtres, faites de papules à disposition souvent linéaires, localisées à la base du cou, aux épaules, aux aisselles et aux plis des coudes [2]. Nous rapportons une forme généralisée, évolutive et compliquée pour souligner la nécessité du diagnostic précoce et du suivi, l'approche multidisciplinaire et la prise en charge des complications.

## Patient et observation

**Figure 1:**
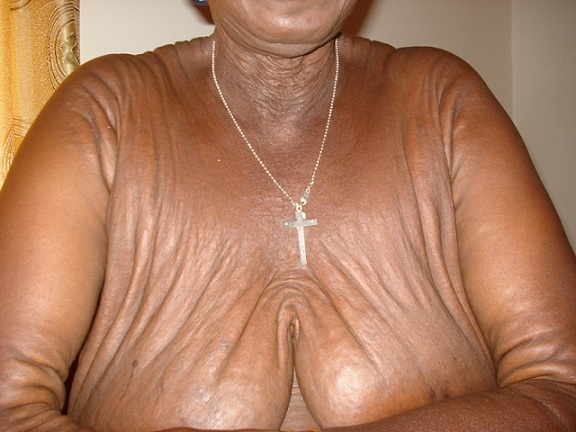
Pseudoxanthome élastique. Aspect en nappes fripées en regard des plis et sur la peau avoisinante

**Figure 2:**
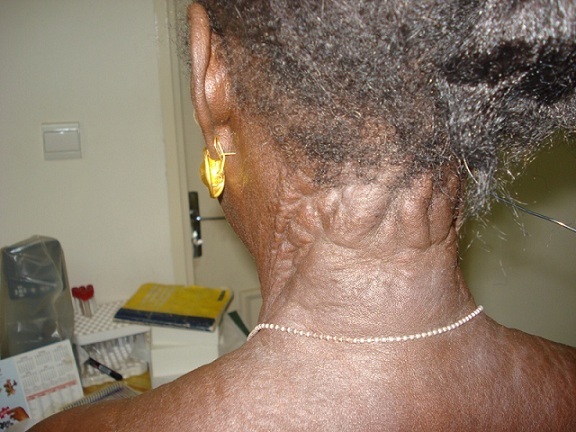
Papules de Pseudoxanthome élastique du cou

**Figure 3:**
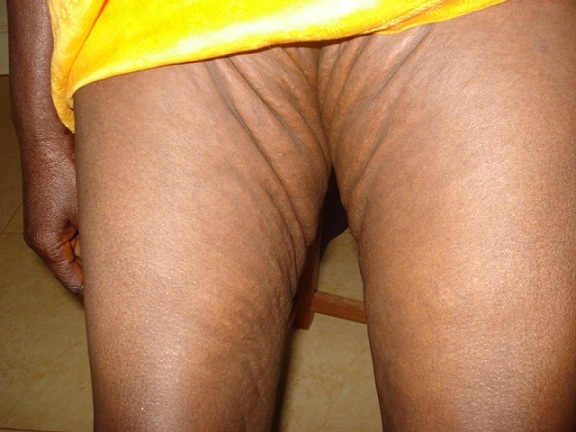
Pseudoxanthome élastique. Aspect en peau de chagrin en regard des plis inguinaux et sur la cuisse

**Figure 4:**
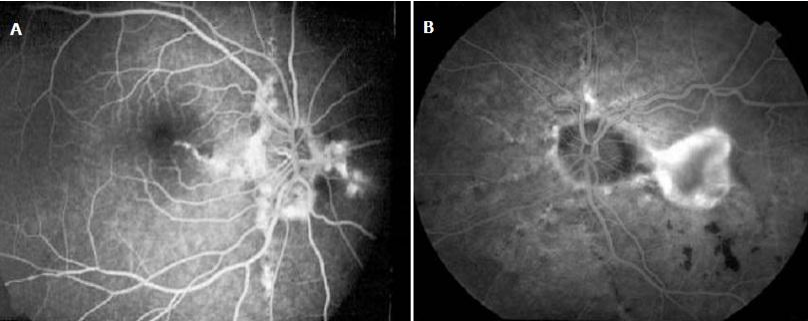
Angiographie à la fluorescéine. Noter les stries angiomateuses des yeux à gauche et les néovaisseaux à droite chez notre patiente

Une femme de 51 ans, ménopausée, avait depuis son enfance des papules hypopigmentées progressivement extensives et confluentes au niveau du cou et des creux axillaires. Un diagnostic précis n'avait jamais été évoqué et la patiente s'en était accommodée. A l'occasion d'une consultation pour une baisse de l'acuité visuelle, elle a été référée par son ophtalmologue en dermatologie pour un avis spécialisé. Les antécédents familiaux n’étaient pas bien précisés mais la patiente avait des problèmes cutanés depuis la petite enfance sans signes fonctionnels. Il n'y avait pas de tare cardiovasculaire, ni métabolique, ni digestive, ni rénale. A l'examen clinique, il n'y avait ni prurit, ni douleurs, ni signes inflammatoires; la patiente avait un bon état général et les signes cutanés étaient représentés par des plaques brunâtres à disposition linéaire, parallèles aux plis, disséminées à tout le corps mais prédominant aux plis et les régions adjacentes; la peau avait un aspect de nappes fripées en “peau de chagrin” ou de “drapé” par coalescence progressive des papules (Figure 1, Figure 2, Figure 3, Figure 4). A la palpation, la peau a perdu de son élasticité. Aucune lésion muqueuse n'a été observée. L'histologie d'une papule montrait des amas de fibres élastiques formant des nodules bien limités. L'examen réalisé en ophtalmologie avait trouvé une baisse de l'acuité visuelle, un segment antérieur normal, une tension oculaire de 13 pour les deux yeux et des stries angioïdes compliquées de néovaisseaux choroïdiens aux deux yeux à l'angiographie à la fluorescéine. L’écho-Doppler cardiaque et la fibroscopie digestive haute étaient normales; il n'y avait pas d'atteinte vasculaire et digestive. La patiente a reçu des informations sur sa maladie, une psychothérapie de soutien et un calendrier de surveillance et confiée en ophtalmologie où elle fut traitée par photocoagulation au laser des néovaisseaux à gauche.

## Discussion

Notre cas est particulier parce qu'il survient chez une femme noire de la cinquantaine avec des lésions cutanées de survenue précoce, étendues progressivement à tous les plis et à la peau environnante, associées à une atteinte oculaire. En 1881, Rigal a été le premier à décrire les lésions cutanées du PXE sous le terme de xanthélasma diffus. En 1929, l'ophtalmologiste Ester Grönblad et le dermatologue James Strandberg ont décrit indépendamment la fréquente association entre lésions cutanées et stries angioïdes [3]. Ce cas confirmait cette observation. Sur peau noire, l'aspect clinique est sensiblement différent, tout en restant évocateur: plus que les classiques papules jaunâtres, on observe dans les localisations habituelles (faces latérales du cou, aisselles, plis des coudes) des placards brunâtres à la consistance cartonnée, prenant volontiers un aspect de « drapé » selon Mahé [4]. Les signes cutanés chez notre patiente confirmaient cette assertion (Figure 1, Figure 2, Figure 3, Figure 4) avec, cependant, des localisations peu classiques diffuses et évolutives; dans notre cas, elles s’étendaient au tégument adjacent, donnant un aspect généralisé. Les papules sont de dimensions variables, parfois confluentes donnant à la palpation l'impression de « velours mouillé » comme cela été observé par Le Saux et Mahé [3, 4]. Ces lésions posent aux patients un problème esthétique parfois majeur. Il existe histologiquement une élastorrhexie des fibres élastiques [5]. Les atteintes extra cutanées font toute la gravité de cette maladie; ce sont surtout les atteintes oculaires pouvant conduire à la cécité, mais aussi les atteintes vasculaires avec athéromatose et rupture des artérioles responsables de saignement [5]. Chez notre patiente, il s'agissait d'atteinte vasculaire oculaire à type de stries et de néovaisseaux pouvant conduire à la cécité. Les stries angioïdes oculaires sont fréquentes, mais non spécifiques sur peau noire: en effet, de telles lésions sont rencontrées au cours de la drépanocytose selon Alabi et al. [6]. Il faut craindre également les accidents d'occlusion vasculaire [5]. Le cardiologue est ainsi un acteur incontournable de la surveillance clinique habituelle; notre patiente y a subi un examen clinique et paraclinique (écho-Doppler cardiaque). Les diagnostics différentiels se posent avec l’élastolyse dermique ou papulose cervicale blanche due à une disparition focale des fibres élastiques du derme superficiel sans élastorrhexie; le PXE se différentie aussi avec la peau citréine du cou qui est une élastose actinique du sujet âgé; il se fera également avec les PXE exogènes où les fibres élastiques sont altérées par le contact avec un produit chimique [1, 7]. Il importe de signaler que les lésions cutanées peuvent être absentes dans quelques très rares cas, les atteintes cardiovasculaires dominant le tableau clinique [8].

En Afrique Subsaharienne, le retard à la consultation est très fréquent et les patients viennent à un stade de complications. Au plan thérapeutique dermatologique, la chirurgie plastique n'est pas indiquée en raison de l’étendue des lésions et du fait qu'il n'y a pas de retentissement psychologique chez notre patiente. Les seules données pronostiques globales disponibles sont celles fournies par McKusick qui avance le chiffre de 50% de mortalité due à la maladie avant 50 ans [9]. Le pronostic est donc favorable chez cette patiente de 51 ans.

## Conclusion

Le PXE nécessite une approche multidisciplinaire pour un conseil génétique, pour la prise en charge des lésions avérées et pour la surveillance associée à l'autosurveillance des complications vasculaires. Aucun traitement spécifique n'est disponible à ce jour.
